# Supplementary figures and images for: How public reaction to disease information across scales and the impacts of vector control methods influence disease prevalence and control efficacy
Source: PLoS Comput Biol. 2021 Jun 28;17(6):e1008762. doi: 10.1371/journal.pcbi.1008762 (PMC8270472; doi:10.1371/journal.pcbi.1008762)

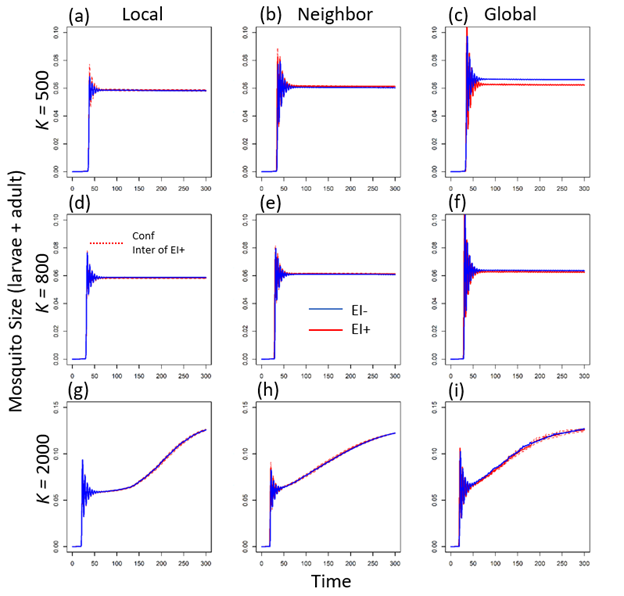

Supplement: S1 Fig — The blue lines indicate the dynamics in the absence of early intervention while the red lines show the mosquito size in the presence of early intervention. (TIFF) [file pcbi.1008762.s001.tiff]

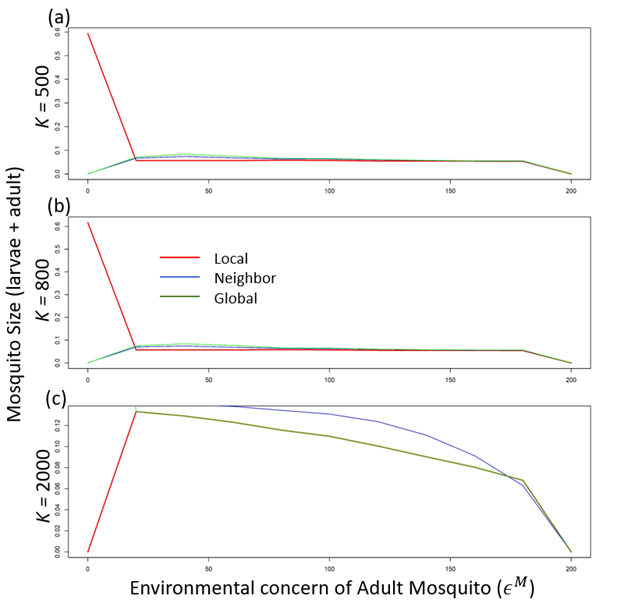

Supplement: S2 Fig — The mosquito population size (larvae + adult) at equilibrium (at time 300) along with the change of environmental concerns on adult mosquito (ϵM) under the influences of information scale (i.e., local, regional, and global) and mosquito breeding capacity. The red lines indicate local scale, the blue lines are for region-scale information, and the green lines are for global scale. (TIFF) [file pcbi.1008762.s002.tiff]
