# Supplementary material for: How public reaction to disease information across scales and the impacts of vector control methods influence disease prevalence and control efficacy
Source: PLoS Comput Biol. 2021 Jun 28;17(6):e1008762. doi: 10.1371/journal.pcbi.1008762 (PMC8270472; doi:10.1371/journal.pcbi.1008762)
Supplement: S1 Appendix — (DOCX) [file pcbi.1008762.s003.docx]

**Appendix The vector sizes under combinations of the scale of disease information and mosquito breeding capacity**

To better understand the potential mechanisms that could influence the average human infections per patch (Fig 2), here we also plot the vector size under three scale levels of disease information and three levels of mosquito breeding capacity.

Under the fixed, equal environmental concerns for both mosquito larvae and adult, mosquito population size slightly increases with the increase of the scale of disease information, but not too much (see the trend from left to right columns in S1 Fig). The vector population size increases with mosquito breeding capacity (see the trend from top to bottom panels in S1 Fig). This is because less restriction on the maximum population size, the more likely the population is to grow. The early intervention (i.e., control actions starting before the first case of local infection) could significantly reduce mosquito population size under global disease information and lower mosquito breeding capacity (S1c and S1f Fig).

With the different combinations of environmental concerns on both air- and water-based control, vector population size at equilibrium is generally smaller at the two extremes of the concerns (i.e., large larval but small adult control vs. small larval but large adult control). However, when the concern of air-based/ or epidemiological control is very small and mosquito breeding capacity is also relatively small (*K =* 500 and 800), vector population size under local-scale disease information is larger compared to the other two scales of information (see the left side of S2a and S2b Fig). Under large breeding capacity (e.g., *K =* 2000 in S2c Fig), vector size under region-scale information is highest when adult concern is relatively large.
