# Supplementary material for: How public reaction to disease information across scales and the impacts of vector control methods influence disease prevalence and control efficacy
Source: PLoS Comput Biol. 2021 Jun 28;17(6):e1008762. doi: 10.1371/journal.pcbi.1008762 (PMC8270472; doi:10.1371/journal.pcbi.1008762)
Supplement: Attachment — Submitted filename: plos_review_3_8.docx [file pcbi.1008762.s004.docx]

Jiao et al. present an interesting theoretical analysis of the relationship between disease information at different spatial scales and vector borne disease dynamics. They provide a generally clear description of their modified Ross-MacDonald model framework and raise a number of points worth considering for public-facing organizations tasked with implementing vector-control strategies.

I have quite a few, but all relatively minor, comments:

**Introduction**:

- Lines 46-47: Please clarify that *Aedes aegypti* is the primary vector of Zika virus and/or provide a reference for this statement.
- Lines 53-54: Newspapers and television are not typically thought of as social media, and the references given focus on traditional social media platforms. If this study is more generally interested in a variety of media as sources of disease information, consider revising this language and adding more additional references.
- Lines 54-58: Do these definitions of spatial scales relate to explicit measures of distance (i.e. is a “local area” meant to represent 1 km^2^, for instance)?

**Methods**:

- Consider including a model diagram or some other visual representation of information and human movement between patches (perhaps in the supplementary material if there is not room in the main text).
- Lines 117-118: Please rephrase or clarify your statement “with initial nodes…”
- Lines 142-143: The estimated incubation period for Zika virus is known to be 3-14 days (Krow-Lucal et al, “Estimated Incubation Period for Zika Virus Disease,” no affiliation with the reviewer). Why was an SIR model framework chosen, rather than SEIR?
- Line 144: Please add *m* and its definition to the table of parameters.
- Lines 145 and 147: What is the relationship between w^H^ and δ? Individuals appear to die at rate w^H^ but are added to the D compartment at rate δ.
- Lines 159-162: Using I to describe both infection and information (apologies if this is a misunderstanding) is somewhat confusing to read. Would you consider changing this notation, or incorporating these values into the table of parameters along with an explanation?
- Line 171: Could you define strength reinforcement here?
- Line 175: It might be helpful to specify the levels of mosquito breeding and why they were chosen.
- Line 184 (Table 2):
  - Could you provide a slightly more descriptive explanation for the composite rate δ? Its meaning is not intuitive.
  - Could you clarify the units/measures of control for Ɛ^M^ and Ɛ^L^?

**Results**:

- Lines 194-196: The difference/relationship between disease information, infection, I^H^, and I^H^_scal_ is still unclear, and might benefit from some rewording or a more explicit definition.
- Line 209: What are the three levels of mosquito breeding capacity and why were they chosen?
- Lines 215-216: Could you please reference a specific subplot here?
- Lines 219-220: This generalization only holds true for subplots A-C. Could you comment further on the trends visible in subplots D-I?
- Lines 229-230: Please reference specific subplots here.
- Line 231: Should Figure 2 be referenced here?
- Line 235: Should this reference Figure 3 rather than Figure 2?
- Figures 1-3, S1, S2: Language in the text alternates between “neighbor” and “regional” to describe the intermediate level of disease information. Perhaps consider using one of these consistently throughout.
- Line 250: Am I correct in assuming that Ɛ^M^ is the x-axis in Figure 4? If so, it might be helpful to label it as such and define the meaning of different x-values explicitly in the caption; this was not intuitive.
- Line 254: What does full environmental control mean?
- Line 275: “Sharp increase” seems like a strong statement, given that the y-scale on the inset graphs are very small. Perhaps moderate this claim or contextualize it.
- Line 279: What does middle concern level mean?
- General:
  - Consider adding more quantitative measures of the relationships depicted here. Perhaps a sensitivity analysis of input parameters related to disease transmission and mosquito scenarios could achieve this?
  - Language around concern and control levels is not consistent. Please standardize these terms and provide explanations/definitions for them.

**Discussion:**

- Lines 291-292: Is this statement based on a figure or a reference?
- Lines 295-296: The final phrase in this sentence is incomplete and its meaning unclear.
- Lines 301-302: Could you further justify this claim and perhaps define strength reinforcement?
- Line 308: If these are the concern preferences, could you maintain this language throughout? Later in this same paragraph, concern preferences are described as water- or air-based too, which seems unnecessarily confusing for the reader.
- Lines 315-316: The meaning of the statement referencing Figure S2 is not clear, please rephrase.
- Line 316: What is the fixed concern situation?
- Lines 320-335: This paragraph reads much like a results section. Could you possibly revise this paragraph to further explore the implications of your findings?
- Lines 337-338: Could you be more specific about the dynamics and assumptions you are referring to?
- Lines 351-353: Are there any real-world examples of local governments doing this?
- Lines 355-356: The claim that public reaction will decrease overall control efficiency seems too strong given the theoretical nature of this study and very specific settings considered. Perhaps consider moderating this language.
- General:
  - Consider adding a discussion of the limitations of this study, such as the exclusion of seasonal temperature variation might influence outcomes and basing disease information solely on infection counts without consideration of possible heterogeneities in behavioral adaptations.
  - It might be beneficial to further emphasize the ways this study makes novel contributions to existing work on the relationship between disease dynamics and (social) media.

**Figure Legend:**

- Lines 372-373, 378-379, 384-385, 392-393: Exclusion of a patch with average values is repeatedly mentioned in all figure captions but nowhere else. Please add a description of the rationale and methodology for this to the Methods.
- Line 389: Please clarify the meaning and units of “concern” here.

**Figures:**

- Lines 396-398: What confidence level was used to generate the intervals in Figures 1 and 2? Were intervals generated for EI- as well? Consider adding these intervals to the figure, perhaps via shading.
- Line 398: Please adjust the y-axis for Figure 2.g-i so that the entire curves are visible.
- Line 403: It is difficult to read the axis values for the inset plots in Figures 4.b and 4.e. Could you make these more visible?
