# Supplementary material for: How public reaction to disease information across scales and the impacts of vector control methods influence disease prevalence and control efficacy
Source: PLoS Comput Biol. 2021 Jun 28;17(6):e1008762. doi: 10.1371/journal.pcbi.1008762 (PMC8270472; doi:10.1371/journal.pcbi.1008762)
Supplement: Attachment — Submitted filename: Cover Letter and answers to Reviewer-4-29-21.docx [file pcbi.1008762.s005.docx]

**Cover Letter**

Dear Editor,

We thank you very much for the positive support and the opportunity to revise our manuscript " How public reaction to disease information across scales and the impacts of vector control methods influence disease prevalence and control efficacy" for publication in *PLOS Computational Biology*. We also thank the reviewer for the useful and detailed comments that have helped us extend the scope of our study and better convey our messages. We have revised our manuscript accordingly and are here resubmitting the revised version. Below, we respond in detail to the points raised by the reviewer. For clarity, we have extracted and numbered their comments and present them in *italics*; our responses follow and are indented. All co-authors have reviewed and approved the revised manuscript.

Sincerely,

Jing Jiao, on behalf of all authors.

Research Associate

Florida State University

**Responses to Reviewer’s comments**

***Reviewer #1:***

*Q1: Good subject material.*

Thank you for the support.

*Q2: I think that the TITLE should have "model" added to it - either at the end or beginning.*

Thank you for the suggestion. We added “model” in the Key words to indicate that the paper involves mathematic model but keep the title short for the main questions this manuscript tried to answer.

*Q3: Please include in the discussion section commentary on insecticide resistance with regard to the impact of this model - any speculation (possibly before the concluding paragraph)?*

Thanks for the suggestion. We added relevant language in Discussion (see Line 388-390 in the new clean version).

*Q4: Please describe on the Figures (1,2, and 3) what the "Time" interval unit is.*

Thank you for the question. We labelled out units for all variables and parameters in Tables 1 & 2 as well as the y-axis units in all figures. For broader implication, our model here does not specifically define the time interval, so it could be day, month or year etc., depending on the time interval in real systems.

*Q5: Please describe on Figure 4 what the Y-axis units are and what is a "concern level of adult mosquitoes". I was unaware how 1 was different from 200.*

Thank you for the question. We added units of y-axis for this figure (Figure 5 in the new manuscript). We also labelled the exact parameter for “concern level of adult mosquitoes” in the legend (see Line 430 in new clean version).

*Please make the following minor corrections:
Q6: Line 51: remove "s" from "...largely depends..." (largely depend)*

Done. See Line 63 in the new version.

*Q7: Line 54: add "the" between ",or Internet" ("or the Internet")*

This sentence was modified based on Q19 from Reviewer #2 (see Line 65-68 in new version).

*Q8: Line 69: "available breeding environment breeding capacity" - should both those breedings be in that sentence or can one be cut out?*

Thank you for catching this up. We made the change by deleting one “breeding” and rearranged the sentence (see Line 81-82 in the new version).

*Q9: Line 75: mosquitoes do not have 2 life stages, they have 4.*

Thank you for the comment. It is indeed true that mosquito could have more than 2 life stages. However, the main goal for mosquito life stages in this manuscript is to study the two common mosquito control strategies – water- (at larval stage), and air-based (adult stage) mosquito control. Including more life stages is not necessary and we believe it would not qualitatively change the results.

*Q10: Line 76: remove "the" from "live in the standing water".*

Done. See Line 88 in the new version.

*Q11: Line 81: reduce "contamination based that are specific to the type" to "contamination specific to the type"*

Done. See Line 93 in new version.

*Q12: Line 86: remove "concerns" in "This tradeoff concerns can..."*

Done. See Line 98 in new manuscript.

*Q13: Line 87-88: Cite the statement: "Larval control has proven to be very effective to control mosquito population" and give the stipulation.*

Thank you for the suggestion. We added language to address larval control on mosquito population and cited one of our previous work (Schwab et al. 2019). See Line 99-100 in the new clean version.

*Q14: Line 91: insert "the" in "...control of adult mosquito..." to read "...control of the adult mosquito..."*

Done. See Line 103 in new version.

*Q15: Line 337: insert ")" after "(46)" to close the parenthetical statement started in line 336.*

Done. See Line 350 in new version.

*Multiple concerns:
Q16: Please elaborate on the statement in line 350-351: more expensive pesticides would expect lower efficacy... ? I would not necessarily agree with that statement - is there an assumption not discussed regarding cost of control measures?*

Thank you for the question. We indeed addressed the assumption of “local economy” at Line 369. Here we further added language to make the statement clearer (see Line 382-383 in the new clean version).

***Reviewer #2***

*Q17: Jiao et al. present an interesting theoretical analysis of the relationship between disease information at different spatial scales and vector borne disease dynamics. They provide a generally clear description of their modified Ross-MacDonald model framework and raise a number of points worth considering for public-facing organizations tasked with implementing vector-control strategies.*

Thank you for the support.

*I have quite a few, but all relatively minor, comments:*

*Introduction:*

*Q18: Lines 46-47: Please clarify that Aedes aegypti is the primary vector of Zika virus and/or provide a reference for this statement.*

Thanks for the suggestion. We made the change accordingly (see Line 59 in the revised version).

*Q19: Lines 53-54: Newspapers and television are not typically thought of as social media, and the references given focus on traditional social media platforms. If this study is more generally interested in a variety of media as sources of disease information, consider revising this language and adding more additional references.*

Thanks, we made the change (see Line 65-67 in the new manuscript).

*Q20: Lines 54-58: Do these definitions of spatial scales relate to explicit measures of distance (i.e. is a “local area” meant to represent 1 km^2^, for instance)?*

Thank you for the question. Here, we do not specifically define the distances of those spatial scales. The main goal is to point out the range difference between the distance social media can reach and the areas people can physically resident in. The exact distance can vary depending on the study system, e.g., a city or a country. Here we added a “diverse” for the spatial scales to clarify our point (Line 67 in the revised version).

*Methods:*

*Q21: Consider including a model diagram or some other visual representation of information and human movement between patches (perhaps in the supplementary material if there is not room in the main text).*

Thank you for the suggestion. We added a plot to represent the information in this study (see Fig. 1 in the new manuscript).

*Q22: Lines 117-118: Please rephrase or clarify your statement “with initial nodes…”*

Here we rephrased the statement to address how the patch structure in the study was generated by scale-free algorithm (see Line 129-130 in the revised version).

*Q23: Lines 142-143: The estimated incubation period for Zika virus is known to be 3-14 days (Krow-Lucal et al, “Estimated Incubation Period for Zika Virus Disease,” no affiliation with the reviewer). Why was an SIR model framework chosen, rather than SEIR?*

Thank you for pointing this out. We agreed that there are studies about Zika virus that applied SEIR model although some studies, including our study, used SIR model structure. In this study, we mainly focus on the mismatch of the spatial scales where people take control actions vs where the infected information is obtained. This scale mismatch will not be affected by whether including incubation period. Therefore, we think that no matter which model structure, either SEIR or SIR, is applied, the main conclusions in our study will not be qualitatively changed. Additionally, while our study is parameterized in the context of Zika virus, we do hope the abstract lessons learned may be broadly applicable to the management of all mosquito-borne disease, and therefore expect that the precise etiological details for each pathogen are of less importance to the reader than the insight we can provide into the general behavior of such systems, which will not fundamentally change due to the inclusion of an incubation period following exposure.

*Q24: Line 144: Please add m and its definition to the table of parameters.*

Thanks for pointing it out. We added the parameter m in Table 2. See Page 13 in the revised version.

*Q25: Lines 145 and 147: What is the relationship between w^H^ and δ? Individuals appear to die at rate w^H^ but are added to the D compartment at rate δ.*

Thank you for the question. The parameter *w^H^* describes the removal rate of the individuals from class *Infected* (*I*) due to mortality. *Dire illness (D)* categorizes the numbers of severe disease consequence due to infection such as Zika-caused birth defects that increase public demand for disease control, separate from risk of mortality. *δ* indicates the rate from *I* to *D,* evaluating how likely the infected individuals get serious disease outcomes. See Table 2 on Page 10-13 in the revised version.

*Q26: Lines 159-162: Using I to describe both infection and information (apologies if this is a misunderstanding) is somewhat confusing to read. Would you consider changing this notation, or incorporating these values into the table of parameters along with an explanation?*

Thank you for the suggestion. We change the notation to $\mathrm{In}_{\mathrm{scal}}^{H}$ and added itto Table 2.

*Q27: Line 171: Could you define strength reinforcement here?*

Thank you for the question. We indeed defined “strength reinforcement” in Line 80.

*Q28: Line 175: It might be helpful to specify the levels of mosquito breeding and why they were chosen.*

We indeed specified the reason why we chose different levels of mosquito breeding capacity in Line 81 to 84. Here we also added the three levels for the following simulation (see Line 188-189 in the revised version).

*Q29: Line 184 (Table 2):*

- *Could you provide a slightly more descriptive explanation for the composite rate δ? Its meaning is not intuitive.*
- *Could you clarify the units/measures of control for Ɛ^M^ and Ɛ^L^?*

Thank you for the suggestion. We added those parameters and units in Table 2 (see Page 11-12).

*Results:*

*Q30: Lines 194-196: The difference/relationship between disease information, infection, I^H^, and I^H^_scal_ is still unclear, and might benefit from some rewording or a more explicit definition.*

Thank you for the comments. We added more language to address the relationship of disease information across scales (see Line 207-210 in the revised clean version).

*Q31: Line 209: What are the three levels of mosquito breeding capacity and why were they chosen?*

See the response to Q28.

*Q32: Lines 215-216: Could you please reference a specific subplot here?*

Yes. We added the subplot numbers here (Line 234 in the new manuscript).

*Q33: Lines 219-220: This generalization only holds true for subplots A-C. Could you comment further on the trends visible in subplots D-I?*

Yes, this trend holds true under all three mosquito carrying capacities: the values of red lines decrease from Fig.4 a-c, d-f and g-i. See Line 239-240 in the new clean version.

*Q34: Lines 229-230: Please reference specific subplots here.*

Done (Line 249 in revised clean version).

*Q35: Line 231: Should Figure 2 be referenced here?*

Yes, we added the reference (now it is Fig. 3 on Line 250 in the new clean version).

*Q36: Line 235: Should this reference Figure 3 rather than Figure 2?*

Yes, we made the change (now it is Fig. 4 on Line 254-255). Thank you for pointing it out.

*Q37: Figures 1-3, S1, S2: Language in the text alternates between “neighbor” and “regional” to describe the intermediate level of disease information. Perhaps consider using one of these consistently throughout.*

Thank you for the suggestion. We use “regional” scale as disease information in the whole text. “Neighbor” is only used to explain the “regional” disease information.

*Q38: Line 250: Am I correct in assuming that Ɛ^M^ is the x-axis in Figure 4? If so, it might be helpful to label it as such and define the meaning of different x-values explicitly in the caption; this was not intuitive.*

Thank you for this suggestion. We here clearly labeled the x-axis as “environmental concern on adult mosquito ($\epsilon^{M}$)”and addressed it clearly in the caption (see Fig. 5 and Line 430 in the new clean version).

*Q39: Line 254: What does full environmental control mean?*

Sorry for the confusion, here we mean the environmental concern only exists in mosquito larvae (i.e., $\epsilon^{L}=200, \epsilon^{M}=0$) or adult ($\epsilon^{L}=0, \epsilon^{M}=200$). The total environmental concern equals to 200 (i.e., $\epsilon^{L}+\epsilon^{M}=200$). See Line 274-275 and the definition of total environmental concern at parameters $\epsilon^{L}$ and $\epsilon^{M}$ in Table 2.

*Q40: Line 275: “Sharp increase” seems like a strong statement, given that the y-scale on the inset graphs are very small. Perhaps moderate this claim or contextualize it.*

Thanks, we took off the “sharp” (see Line 295 in the revised version).

*Q41: Line 279: What does middle concern level mean?*

Thanks for asking. We mean the level at $\epsilon^{M}=100$. We added language on Line 297, 300 in the revised version.

*Q42: General:*

- *Consider adding more quantitative measures of the relationships depicted here. Perhaps a sensitivity analysis of input parameters related to disease transmission and mosquito scenarios could achieve this?*
- *Language around concern and control levels is not consistent. Please standardize these terms and provide explanations/definitions for them.*

Thank you for all the suggestions. We did verify the general patterns by modifying the parameter values. We also made changes according to your detailed comments, which largely improve the manuscript. While we have not included a formal sensitivity analysis, this is because our work builds on previously published studies (e.g., Schwab *et al.* 2018, 2019 in the manuscript) of the same model that show the model is qualitatively robust to small deviation in vector ecological and disease etiological rates and in this paper, we choose to focus on the insight generated from broad qualitative behaviors of the disease system.

*Discussion:*

*Q43: Lines 291-292: Is this statement based on a figure or a reference?*

This statement is mainly from the foundational logic of the system. We believe our study is the first one that focuses on the influence of larger-scale disease information on disease dynamics.

*Q44: Lines 295-296: The final phrase in this sentence is incomplete and its meaning unclear.*

Thank you for pointing this out. We made the change to make the sentence clear (Line 315-319 in the revised clean version).

*Q45: Lines 301-302: Could you further justify this claim and perhaps define strength reinforcement?*

See our answer to Q27. We defined this term in Line 80, but here we readdressed it (see Line 325-327 in the new clean version).

*Q46: Line 308: If these are the concern preferences, could you maintain this language throughout? Later in this same paragraph, concern preferences are described as water- or air-based too, which seems unnecessarily confusing for the reader.*

Thanks for this comment. We now used “adult” and “larval” instead of “water-” and “air-” through the whole paragraph. See Line 337 and 339 in the revised manuscript.

*Q47: Lines 315-316: The meaning of the statement referencing Figure S2 is not clear, please rephrase.*

Thanks for pointing this out. We now changed the label of x-axis of Fig. S2 explicitly, as well as added language for notations in the main text (see Line 341 in the revised clean version).

*Q48: Line 316: What is the fixed concern situation?*

We added language to explain the fixed concern (see the equation in parenthesis at Line 341-342 in the revised clean version).

*Q49: Lines 320-335: This paragraph reads much like a results section. Could you possibly revise this paragraph to further explore the implications of your findings?*

Thank you for the suggestion. This paragraph tends to explain some complicated results by referring to related previous studies. Here we added one summary at the end of this paragraph to highlight the purpose of this paragraph (see Line 360-363 in the revised clean version).

*Q50: Lines 337-338: Could you be more specific about the dynamics and assumptions you are referring to?*

Thank you. We added the language for the assumptions (Line 366-367 in revised clean version).

*Q51: Lines 351-353: Are there any real-world examples of local governments doing this?*

Many real examples are related to COVID-19, AIDS. Here we cited related papers (see citations in Line 387 in the new clean version).

*Q52: Lines 355-356: The claim that public reaction will decrease overall control efficiency seems too strong given the theoretical nature of this study and very specific settings considered. Perhaps consider moderating this language.*

Thank you, we changed “would” to “may” (Line 392 in revised clean manuscript).

*Q53: General:*

- *Consider adding a discussion of the limitations of this study, such as the exclusion of seasonal temperature variation might influence outcomes and basing disease information solely on infection counts without consideration of possible heterogeneities in behavioral adaptations.*
- *It might be beneficial to further emphasize the ways this study makes novel contributions to existing work on the relationship between disease dynamics and (social) media.*

Thank you for the suggestions. We added more language to discuss the potential limits of this study and future work direction (Line 376-390). We also added more language about previous studies about disease dynamics and social media (see Line 309-312 in the revised clean version).

*Figure Legend:*

*Q54: Lines 372-373, 378-379, 384-385, 392-393: Exclusion of a patch with average values is repeatedly mentioned in all figure captions but nowhere else. Please add a description of the rationale and methodology for this to the Methods.*

We added the language at Line 191-192 in the revised version.

*Q55: Line 389: Please clarify the meaning and units of “concern” here.*

See our answer to Q5. We made the change of Fig. 5 caption (see Line 430 in revised clean version).

*Figures:*

*Q56: Lines 396-398: What confidence level was used to generate the intervals in Figures 1 and 2? Were intervals generated for EI- as well? Consider adding these intervals to the figure, perhaps via shading.*

Thank you. We added the 95% CI to the figure captions (Line 415, 421 in the revised version). For EI-, the CI is very narrow and overlapped, so it is not observable.

*Q57: Line 398: Please adjust the y-axis for Figure 2.g-i so that the entire curves are visible.*

Thank you, the black lines have much higher values in human infected cases, so all red and blue lines cannot be observed. Therefore, we showed the y-axis range to show the clear patterns of the red and blue lines, which we mainly focused on in the text.

- *Q58: Line 403: It is difficult to read the axis values for the inset plots in Figures 4.b and 4.e. Could you make these more visible?*

Yes, we made the x- and y-axis values are readable in Fig. 5.

***Reviewer #3:***

*Q59: This paper raises an interesting question, and models fairly simply yet convincingly the impact of public attitudes towards a raising threat by a vector-borne disease such as Zika.*

Thank you for the support.

A few remarks nonetheless:

*Q60: even from the abstract on, the authors focus on "social media" and its impact. Yet, the model does not seem to address specifically this problem. There is no explicit modeling, for instance, of a "viral" transmission of information on a social media platform. Here, the focus is mostly on public attitudes ("reactions" if you will) towards a new epidemics in their area or not that far from them. But these reactions could be driven by anything else (traditional media, rumors etc.), and the model would still be correct.*

Thank you for the suggestion. See our answer for Q19, in which we extended the potential media including traditional ones etc..

*Q61: the "overreaction" term seems unsuitable for the situation described. Individuals are indeed correct in assuming that their situation is connected to the situation in an area close to them. Talking about "overreaction" would then imply that there is a proper level of reaction, i.e. an adequate value for "risk aversion".*

Thank you for the question. “Overreaction” here aims to describe the situation local disease control is based on the infected number in larger spatial scale (i.e., the disease information is “over” from the larger scale vs. local scale). We do not intend to discuss whether this reaction is at a proper level or not. To keep this clear, we changed “overreaction” to “sensitive reactivity” (see 32-33, 36, 313, 387 and 396 in new clean version).

*Q62: the conclusion regarding impact on public policy seems overly optimistic, as the the realism of this model seems still precarious. A few assumptions are still very strong, some mechanisms are ignored (no explicit way to determine whether "overreaction" will occur in a certain epidemic or not; no feedback between actions taken by the government / the population and the attitudes towards the epidemic ; no sens of the politisation of a crisis - whatever the authorities recommend is perfect/disastrous depending on your political opinion etc.)*

*This article is however a good first step towards a better understanding of these situations.*

Thank you for this comment. This model is indeed a first step to bring attention about this “overreaction” due to disease information from larger scale. Further studies could include government actions and political opinion etc.

***Reviewer #4:***

*Q63: The overall messaging of the study is interesting. Calculations and conclusions drawn from model agree with underlying theme of the work.*

Thank you for the support.

*This thesis is nicely argued, I have some minor points:

Q64: The omega (line 145) parameter in the main equations 1-9 is not explained in the text. Perhaps that’s a typo and is meant to be delta? If not then I do not see any transfer rates from infectious compartment (I_H)to seriously infected compartment (D^H). Some typos in Eqns 7-9 probably. If not for the typos then the notation for control parameters and control change in larvae/adult mosquitoes is confusing. Notation of total control effort line 170 with equations 8-9 could be better, specially given hat small 't' is used for time, Again some inconsistency with notation between parameters in the table and in equations--makes for a difficult reading.*

Thank you for the suggestions. We listed out all parameters, including their descriptions, values/units and citations if any in Table 1&2. $\omega^{H}$ is the disease-induced mortality in Table 2. The transfer rate from $I_{j}^{H}$ to $D_{j}^{H}$ is the composite rate $\delta$ in Table 2 as well. We hope the notations are clear in the new version.

*Q65: Equations in line 193 could be written in separate lines perhaps, right now equation numbering has crept into the main text. Make the notation of I_scale (from lines 159 to 153) consistent with the notation used for local, regional and global scale used at line 193, Eqs. 13 and 14.*

Thank you for the suggestion. We added ${In}_{scal}^{H}$ at three scales (see Line 211-212 in new version) to the notation at Line 171-173. We still keep the two eqn.s 13-14 (at Line 211-212) inside the text instead of separate lines because we think these two eqn.s are within one sentence.

*Q66: Figure 4(c) and the text explaining it is currently not consistent. There is a dip in 4c at local scale, could that be explained somehow? Also the legends, labels, and captions need to be improved for all figures. It is very hard to understand them in the current format.*

Thank you for the suggestion. We indeed explained the dip in Fig. 4c, which is Fig. 5 in the new version. See Line 294-300 in new version.

*Q67: The results subsections could use better titles, my suggestions would be: Early intervention and delayed intervention. I leave to the authors to come up with better titles.*

Thank you. We consider the two subsections of first section in Results by including and excluding early intervention. There is no delayed intervention. We modified the subtitles to address the statement clearer (see Line 222 and 243 in new clean version).
